# Intestinal Flora Disruption and Novel Biomarkers Associated With Nasopharyngeal Carcinoma

**DOI:** 10.3389/fonc.2019.01346

**Published:** 2019-12-06

**Authors:** Haiye Jiang, Jian Li, Bin Zhang, Rong Huang, Junhua Zhang, Ziwei Chen, Xueling Shang, Xisheng Li, Xinmin Nie

**Affiliations:** ^1^Clinical Laboratory, Third Xiangya Hospital, Central South University, Changsha, China; ^2^Department of Blood Transfusion, Third Xiangya Hospital, Central South University, Changsha, China; ^3^Department of Anatomy and Neurobiology, Biology Postdoctoral Workstation, School of Basic Medical Sciences, Central South University, Changsha, China

**Keywords:** biomarker, nasopharyngeal carcinoma, familial, sporadic, intestinal flora, 5-HT

## Abstract

**Background:** Nasopharyngeal carcinoma (NPC) is a malignant nasopharyngeal disease with a complicated etiology that occurs mostly in southern China. Intestinal flora imbalance is believed to be associated with a variety of organ malignancies. Current studies revealed that the destruction of intestinal flora is associated with NPC, and many studies have shown that intestinal flora can be used as a biomarker for many cancers and to predict cancer.

**Methods:** To compare the differences in intestinal flora compositions and biological functions among 8 patients with familial NPC (NPC_F), 24 patients with sporadic NPC (NPC_S), and 27 healthy controls (NOR), we compared the intestinal flora DNA sequencing and hematological testing results between every two groups using bioinformatic methods.

**Results:** Compared to the NOR group, the intestinal flora structures of the patients in the NPC_F and NPC_S groups showed significant changes. In NPC_F, *Clostridium ramosum, Citrobacter* spp., *Veillonella* spp., and *Prevotella* spp. were significantly increased, and *Akkermansia muciniphila* and *Roseburia* spp. were significantly reduced. In NPC_S, *C. ramosum, Veillonella parvula, Veillonella dispar*, and *Klebsiella* spp. were significantly increased, and *Bifidobacterium adolescentis* was significantly reduced. A beta diversity analysis showed significant difference compared NPC_F with NOR based on Bray Curtis (*P* = 0.012) and Unweighted UniFrac (*P* = 0.0045) index, respectively. The areas under the ROC curves plotted were all 1. Additionally, the concentrations of 5-hydroxytryptamine (5-HT) in NPC_F and NPC_S were significantly higher than those of NOR. *C. ramosum* was positively correlated with 5-HT (rcm: 0.85, *P* < 0.001). A functional analysis of the intestinal flora showed that NPC_F was associated with Neurodegenerative Diseases (*P* = 0.023) and that NPC_S was associated with Neurodegenerative Diseases (*P* = 0.045) as well.

**Conclusion:** We found that NPC was associated with structural imbalances in the intestinal flora, with *C. ramosum* that promoted the elevation of 5-HT and opportunistic pathogens being significantly increased, while probiotics significantly decreased. *C. ramosum* can be used as a novel biomarker and disease prediction models should be established for NPC. The new biomarkers and disease prediction models may be used for disease risk prediction and the screening of high-risk populations, as well as for the early noninvasive diagnosis of NPC.

## Introduction

Nasopharyngeal carcinoma (NPC) is a head and neck cancer (HNCC) caused by a malignant transformation of the nasopharyngeal epithelium. Based on the International Agency for Research on Cancer, nearly 129,000 new patients of NPC were diagnosed in 2018, and more than 70% of the new patients are from East and Southeast Asia. The NPC incidence rate in China has reached 3/100,000 (1, 2). The early symptoms of NPC are rare and not prominent, so NPC is not easily identified in the early stage; in addition, 70% of patients are at a locally advanced stage at the time of diagnosis (3), one of the major clinical symptoms is cervical lymph node enlargement. Early diagnosis, intervention and treatment are important prognostic factors for NPC patients and can significantly reduce their mortality. The causes of NPC are complicated and diverse and the currently recognized causes include genetic susceptibility, eating habits and Epstein-Barr virus (EBV) infection (4, 5). Multiple studies have reported that NPC shows a characteristic of family aggregation in low-risk populations and southern China (6–8). Epidemiological studies have also shown that people with a first-grade NPC family history are 4–20 times more likely to develop NPC than those who do not have a family history (9–11). The intestinal flora is a symbiotic microflora that inhabits the human intestinal mucosa (12), and its structural balance plays indispensable roles in various aspects including the immune response, metabolism of carcinogens and nutrient digestion (13, 14). Although there are many possible explanations for the formation of the intestinal flora, it is agreed that the intestinal flora is familial (15, 16), and this feature is probably related to the occurrence of familial tumors. It is worth noting that patients with familial colorectal cancer have similar imbalanced intestinal flora structures (17), which may cause a metabolic disruption of the intestinal metabolites and the disorders of the immune system, leading to cancer. Numerous studies have shown that changes in the intestinal microbiota are significantly associated with colorectal cancer (CRC) (18) and many extraintestinal malignancies (19), such as liver cancer (20), pancreatic cancer (21), melanoma (22), and breast cancer (23), there is a consensus that the dysbiosis of intestinal flora can promote cancer through provoking the magnification of immune response (19); meanwhile, it is found that intestinal microbiota can drive immune T cells proliferation and activation, which leads to the enlargement of cervical lymph nodes (24). Nevertheless, the hidden mechanisms of the interaction among intestinal flora, cancer and immune system remain exploiting completely (19, 25, 26). It is discovered that the dysbiosis of oral microbial structure is associated with HNCC (27), there are many similarities and connections between intestinal flora and oral microbiota (28), thus these discoveries above hinted us that the intestinal microbiota may modulate the development NPC by regulating the immune system. Moreover, a previous study found that radiochemotherapy significantly destroyed the balance of intestinal flora in NPC patients and that probiotic combination could significantly alleviate oral mucositis in NPC patients by improving the intestinal flora (29), which also prompted us that the intestinal flora may play an important role in the progression of NPC. In the meantime, one study reported that native spore-forming bacterium from the human intestinal flora played a major role in elevating the level of 5-HT in plasma from modulating enterochromaffin cells (ECs) (30).

5-HT is an important neurotransmitter and vasoactivator that is mainly synthesized and secreted by the central nervous system (CNS) and ECs, which has functions to regulate neurotransmitters and neuroendocrine (31). A variety of researches have found that 5-HT can stimulate the development and progression of multifarious cancers, such as prostate carcinoma (PC) (32), hepatocellular carcinoma (HCC) (33–35), CRC (36), small-cell lung cancer (SCLC) (37), pancreatic ductal adenocarcinoma (PDAC) (38, 39), cholanfiocarcinoma (40), breast cancer (41), ovary carcinoma (42), and glioma (43) and carcinoids (31) through the 5-HT receptor (5-HTR) subtypes like 5-HT1A, 5-HT1B, 5-HT2A, 5-HT2B etc. What interested us most is that a study has found that 5-HT1B was overexpressed in human NPC samples (44), which reminds us that 5-HT may play a crucial role in NPC development. This has aroused our great interest in research. *C. ramosum* is an anaerobic, Gram-positive spore-forming bacteria that produces immunoglobulin (Ig) A protease, which can be mainly found in intestinal tract (45). A previous study has shown that the metabolites of *C. ramosum* could stimulate the secretion of 5-HT from ECs, which can promote the level of 5-HT in plasma (46). These series of discoveries mentioned above have induced us to get a scenario that intestinal microbiota can promote the secretion of 5-HT to facilitate the progression of NPC.

Current screening methods for NPC include gene sequencing, EBV immunology and EBV DNA (47). However, these invasive methods are costly and time-consuming, so there is an urgent need to develop a new economical and non-invasive detection method for early NPC screening. This objective may be accomplished by using the specificity of the composition of the intestinal flora. Studies have shown that the intestinal flora can be used as a special biomarker for the screening of CRC with a sensitivity of 77.7% and a specificity of 79.5% (48, 49). The family-specific intestinal flora is also expected to be used for NPC screening.

In this study, we recruited familial NPC patients (NPC_F), sporadic NPC patients (NPC_S), and healthy controls (NOR) and performed the 16S rRNA sequencing of their intestinal floras and examined multiple clinical indicators of their blood. We compared the composition and biological functions of the intestinal floras among NPC_F, NPC_S, and NOR through bioinformatic methods, and explored the association between changes in the intestinal flora and NPC_F and NPC_S, elaborated the association that the intestinal flora had an impact on NPC by modulating the secreting of 5-HT, contemporary. We predicted the functions of each flora of NPC patients, aiming to establish the connection between every two groups through analyzing the intestinal flora of NPC patients. This study will lay a foundation for the application of the intestinal flora to the early diagnosis of NPC.

## Materials and Methods

### Recruitment of Volunteers

We recruited 481 NPC patients and staged their tumor status with the American Joint Committee on Cancer (AJCC) 7th edition staging criteria (50). Excluded 243 patients with other diseases, excluded 50 patients who had received anti-tumor treatments, excluded 60 patients who had any drug treatments within 1 month, and excluded 44 patients with family history of any other tumors. Finally, 84 patients who met our requirements remained. And then, eight NPC patients [(46.4 ± 5) years old] with a NPC family history were selected, because the factors such as age, gender and BMI have influence on intestinal microbial structure, 24 sporadic NPC patients [(47.3 ± 3.3) years old] matched with familial NPC patients in age, gender and BMI were selected. At the same time, 87 healthy volunteers were recruited, and 27 healthy volunteers [(47.2 ± 3.4) years old] whose age, gender and BMI were matched with NPC patients were selected as controls.

We randomly recruited the NPC patients and healthy volunteers in the same area in China, who had a parallel dietary background. The recruited NPC group and healthy group were in line with the principle of randomized control, which could avoid various biases and balance the confounding factors including the nutrition and dietary intake (51, 52). The healthy volunteers were defined as those between 18 and 70 years old, with no history of NPC, no family history of NPC, no history of rhinitis, no history of any other diseases (such as hypertension, diabetes, gastrointestinal diseases, and immune diseases), and no smoking or drinking history, who did not receive any antibiotics or treatment 3 months prior to sample collection. None of the NPC patients had rhinitis or had used any antibiotics during the 3 months prior to sample collection, none of the patients had received any kind of treatment since being diagnosed NPC, including radiotherapy and chemotherapy. The study was reviewed by the Ethics Committee of the Third Xiangya Hospital of Central South University. Informed consent was signed by all subjects prior to participation.

### Sample Collection

Fecal samples and blood samples were collected separately from each subject; venous blood collection was performed by professional nurses strictly in accordance with sterile and standardized procedures. Fecal samples were collected by the subjects themselves, before collecting the feces, we would teach the volunteers how to collect the feces, the procedures were as follows: Feces will be excreted on a piece of dry and clean paper, and the middle part of feces will be picked up with the pick stick of sterile feces collector and then placed it into the dry sterile feces collector immediately. After collection, the sterile feces collectors with feces were immediately placed on ice, to ensure the temperature was below 4°C and transported them to the biobank within 1 h, where they were stored at −80°C until DNA extraction (53). Sample collection, packaging, and storage procedures were strictly following the protocols and regulations of the Biobank of the School of Basic Medical Sciences, Central South University.

### Testing of Blood Specimens

#### Detection of Routine Clinical Indicators for Blood

Blood routine test was performed using BC-6800 blood cell analyzer (Mindray, Shenzhen, China) within 8 h after collection of anticoagulated whole blood. Serum samples were tested using the 7600-020 automatic biochemical analyzer (Hitachi, Tokyo, Japan) for the detection of high-sensitivity C-reactive protein (hCRP), total cholesterol (TC), triglyceride (TG), total protein (TP), albumin (ALB), globulin (GLO), albumin to globulin ratio (A/G), total bilirubin (TBIL), blood urea nitrogen (BUN), uric acid (UA), alanine aminotransferase (ALT), fasting blood glucose (FBS), and bile acid (TBA).

#### Detection of 5-HT Concentration for Blood

Human sera were tested for 5-HT using a human 5-HT ELISA KIT (Mlbio, Shanghai, China) according to the manufacturer's instructions. In general, the steps were as follows: The standard and diluted samples were added to the standard well and sample well, respectively, at 50 μL per well. Then, the enzyme-linked reagent was added to each well and incubated at 37°C in the dark for 60 min. After repeated washing, chromogenic reagent was added for color development. In the end, the stop solution was added, and the OD value of each well was measured at the wavelength of 450 nm using a microplate reader (Biotek, USA). The concentration of 5-HT in the sample was calculated according to the standard curve.

### DNA Detection in the Intestinal Microbes

#### DNA Extraction

The total DNA was extracted using the E.Z.N.A.^®^ soil kit (Omega Biotek, Norcross, GA, USA) according to the manufacturer's instructions. The E.Z.N.A.^®^ soil DNA kit permits efficient and dependable extraction of high-quality genomic DNA from various samples, including clinical samples (54, 55). The DNA concentration and purity were determined using a NanoDrop 2000 (Thermo Fisher Scientific, USA). The quality of the DNA extraction was assessed by electrophoresis using a 1% agarose gel. Finally, the 16S V3-V4 variable region was subjected to PCR amplification using the 338F (5′-ACTCCTACGGGAGGCAGCAG-3′) and 806R (5′-GGACTACHVGGGTWTCTAAT-3′) primers.

#### DNA Sequencing

The PCR products were isolated using a 2% agarose gel, purified and quantified. A PE 2^*^300 library was constructed using the purified amplification fragments according to the standard procedure of the TruSeqTM DNA Sample Prep Kit (Illumina, San Diego, USA). Finally, the DNAs were sequenced using the Miseq PE300 sequencer (Illumina, San Diego, USA).

### Processing and Analysis of Sequencing Data

#### Sequence Processing

The amplicon sequence variant (ASV) was obtained after quality control, denoising and the removal of chimeras using the DADA2 method recommended by QIIME2 according to the raw sequence information (FASTQ format) (56). The ASV was compared with the GREENGENES database (57) (the database was aligned to the V3-V4 region according to the 338F/806R primers) and annotated. The ASV with 99% similarity was classified as one Operational Taxonomic Unit (OTU) to obtain the OTU classification information table. The OTUs were classified using the RDP classifier to obtain their numbers at different taxonomic levels.

#### Differential Flora Analysis

We determined the bacteria with differences in abundance among groups and samples using the Kruskal-Wallis, LEfSe and DEseq2 methods (58, 59) and adjusted the *P* value using the Benjamini-Hochberg method (60). In our study, an underestimated false discovery rate (FDR) (*P*-adjust in standard R packages) was used to rectify multiple measurements (61), the rectified FDR known as *P* < 0.1 was considered significant. The threshold value 0.1 for *P*-value was used mostly to be a significant threshold in human microbial genomics, to embrace the crucial taxa with small effect sizes (62–64). Kruskal-Wallis is a nonparametric test with no requirements for distribution and is appropriate for the bacterial flora analysis. To identify differential microbial species among the different groups, we used the linear discriminant analysis effect size (LEfSe) analysis based on the linear discriminant analysis (LDA) and assessed the significant differences with an LDA score >2.0 as the critical value. DESeq2 is a method for the differential analysis of counting data that allows multiple comparisons among groups to find microorganisms that differ significantly between two groups.

#### Alpha Diversity

To determine the abundance and homogeneity of the sample species composition, we calculated the alpha diversity indices including the observed OTUs, Shannon index and Faith's phylogenetic diversity index and compared the differences in the alpha diversity among groups. The observed OTUs was used to determine the abundance of OTUs in the samples, and the Shannon index was used to calculate the homogeneity of the samples. Faith's phylogenetic diversity index was used to calculate the distance from the OTU of each sample to the phylogenetic tree. The rarefaction curve showed the number and/or the diversity of the species and was used to determine the reliability of the sequencing data of the samples that indirectly indicated the species richness in the samples.

#### Beta Diversity

To determine the differences in the microbial community compositions among the different samples, we used the beta diversity index that was based on the Bray-Curtis and Unweighted UniFrac indices for analysis. Bray-Curtis is the most commonly used indicator in ecology that reflects differences among communities and it shows the information of species abundance. The Unweighted UniFrac distance is the distance between samples calculated based on the evolutionary relationship of the species systems that mainly considers the presence or absence of species. These two indices were used for the principal coordinate analysis (PCoA), and the principal coordinate combination with the largest contribution rate was used for graphing (65). To perform a statistical analysis of the PCoA analysis results, the results were subjected to a significant analysis using the nonparametric test analysis of similarities (Anosim).

#### Establishment of an Intestinal Flora Prediction Model

We also used the partial least squares discriminant analysis (PLS-DA) to predict the sample types corresponding to microbial communities (66). PLS-DA is a supervised analytical method that distinguishes groups using mathematical models, ignores random differences within groups and highlights systematic differences among groups. The classification performance among groups by PLS-DA based on intestinal flora markers can be evaluated using the receiver operator characteristic (ROC) curve. The area under the curve (AUC) is closer to 1, indicating that the prediction model obtained by the PLS-DA analysis is reliable, and multiple microorganisms differ in their abundances among groups.

#### Association Analysis Between Intestinal Flora and Clinical Variables

The redundancy analysis method (RDA) (67) was used to analyze the potential association between the intestinal flora and clinical variables based on relative abundances of microbial species at different taxa levels using the R package “vegan” (68), so that the important driving factors that affected the distribution of the flora could be obtained. In the RDA species sorting map, clinical variables were indicated by arrows, where the length of an arrow represented the degree of correlation between the clinical variable and the flora distribution (that indicated the size of the variance). An acute angle between arrows indicated a positive correlation between two clinical variables, and an obtuse angle indicated a negative correlation. Each point represented a species, and the larger the point, the more abundant the species. To determine if a clinical variable was significantly correlated with a microbial community or species, we calculated the Spearman correlation coefficient between the microbial species and clinical variables, which is shown in the correlation heat map.

#### Intestinal Flora Function Prediction

We used PICRUSt software (69) to predict the function of intestinal flora based on the 16S species information and KEGG function information. The principle of PICRUSt is to deduce the microbial genomic function region from the 16S rRNA sequence information, the sequenced 16S rRNA information is corresponded to the genomic functional prediction spectrum in the GREENGENES database to predict the metabolic function of the microbiota (69, 70). According to the depth of annotation, KEGG function can be annotated at three levels. In addition, we performed the principal component analysis (PCA), Dunn test and Duncan analysis for the predicted functions, and corrected the *P*-values using the Bonferroni method (71). Through PCA we calculated the principal components with the largest contribution to represent most of the variation of the samples (72). Dunn test and Duncan analysis are two kinds of significant analysis methods, the Dunn test is mainly used for pairwise comparison, while the Duncan analysis is used for multiple comparison. The results were displayed in a PCA map of the prediction function, a bar chart of the KEGG pathway at the second level and a bar chart of significant KEGG pathway comparing the three groups.

According to the PLS-DA analysis, the three distinct clusters indicated that the intestinal floras of the NPC_F, NPC_S, and NOR groups were clearly differentiated into three independent clusters, indicating that the composition of intestinal flora was significantly different among the three groups. All the AUCs were 1, suggesting that the prediction model established based on the detected differential genus was perfect by using the PLS-DA analysis and that the intestinal flora related to the two groups of NPC were strong prediction factors and could be used as risk factors for NPC. For example, *C. ramosum* could be used as a strong prediction factor for NPC and is likely to be developed as a biomarker for high-risk populations and NPC.

To determine whether the structural differences among the three groups of intestinal flora corresponded to functional changes, we used PICRUSt to perform a functional prediction analysis of the 16S sequences and performed a PCA analysis of the predicted functions and a comparative analysis of the different KEGG-pathway levels.

## Results

### Volunteer Characteristics

**Figure 1:**
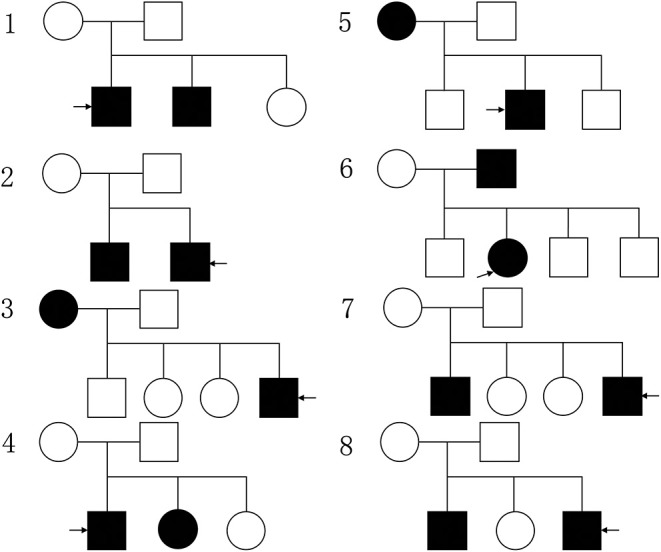
The genetic map of familial NPC patients, the circle represents female, the square represents male, the black pattern represents the person with NPC, while the blank one means the healthy person; the pattern indicated by the arrow represents the patient participating in the study.

The demographic and clinical characteristics of 8 familial NPC patients, 24 sporadic NPC patients and 27 healthy volunteers are shown in Supplementary Table 1. The tumor stage of recruited 84 NPC, and 8 familial NPC and 24 sporadic NPC patients included in the study is shown in Supplementary Table 6. There were no significant differences between three groups of the basic characteristics in age, gender, race etc. The genetic map of familial NPC patients is presented in Figure 1.

### Hematology Detecting Results

**Figure 2:**
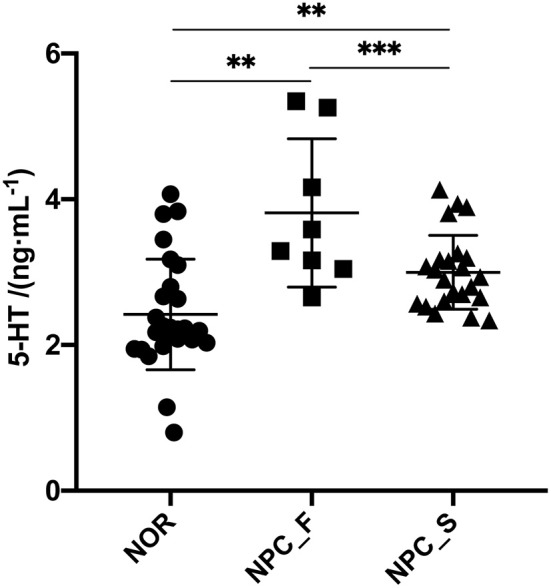
The concentration of 5-HT in sera of the healthy controls (NOR), familial NPC patients (NPC_F), and sporadic NPC patients (NPC_S) groups, ^**^P < 0.01, ^***^P < 0.001.

There are many differences between a variety of clinical indicators by statistical analysis among groups of multiple hematology test results (Supplementary Table 2). It's worth noting that the concentration of hCRP was significantly different between NPC: NOR (*P* = 0.0047), indicating that hCRP concentration was significantly higher in NPC patients than NOR. The concentration of 5-HT in serum was determined by ELISA (Figure 2). The concentration of 5-HT in serum of NOR group was (2.421 ± 0.300) ng/mL, for NPC_S group was (2.986 ± 0.207) ng/mL, and for NPC_F group was (3.813 ± 0.850) ng/mL. The concentration of 5-HT in NPC_F group was significantly higher than NOR (*t* = 3.499, *P* = 0.0014) and NPC_S (*t* = 3.841, *P* = 0.0003), and the concentration of 5-HT in NPC_S group was significantly higher than the NOR group (*t* = 2.997, *P* = 0.0046).

### DNA Sequencing Results of Intestinal Flora and Statistical Analysis

#### Species Abundance and Diversity

**Figure 3:**
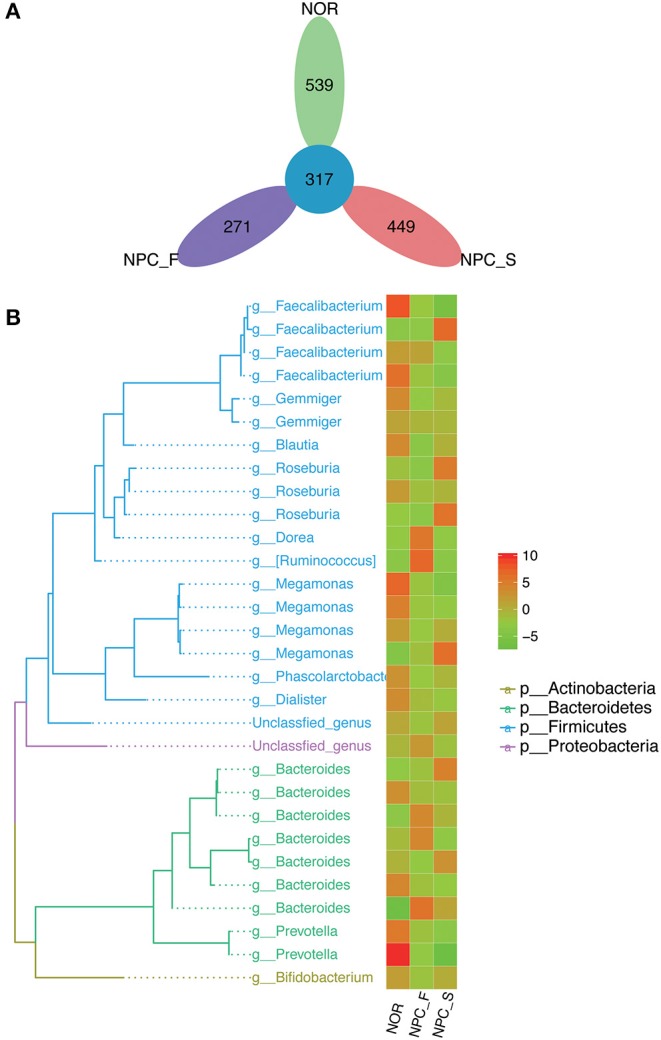
Comparison of OTUs and phylogenetic tree map, **(A)** petal diagram based on OTUs of the healthy controls (NOR), familial NPC patients (NPC_F) and sporadic NPC patients (NPC_S) group. **(B)** Phylogenetic tree map, the phylogenetic tree on the left consists of nodes and branches, the different color of the branches represents the classification at different phylum levels, each terminal node represents an OTU, the corresponding classification of the OTU at the genus level is showed at the end of the branch. The heat map on the right clusters the standardized abundance of the genus corresponding to the left, through value-color gradient, the redder the color, the larger the value, the richer the abundance (phylum to genus: p, phylum; g, genus). Abundance standardization: the absolute abundance of each sample minus the mean absolute abundance of the genus and divided by the standard deviation; the normalized mean abundance is 0 and the standard deviation is 1.

**Figure 4:**
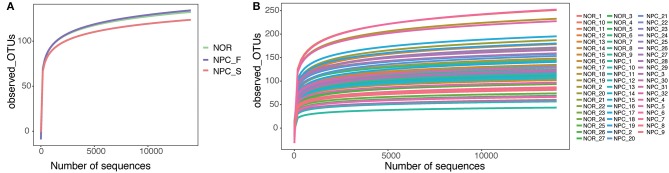
Dilution curve in observed_OTUs, **(A)** Dilution curves of the healthy controls (NOR), familial NPC patients (NPC_F), and sporadic NPC patients (NPC_S) groups. **(B)** Dilution curves of all of the samples.

A total of 3,200,797 available raw readings were obtained from all 59 samples, with an average reading of 54,251 ± 4,044 per sample. After CD-HIT clustering and NAST alignment, 1,828,692 unique representative sequences were generated and total of OTUs was 2,894. Petal diagram (Figure 3A) and phylogenetic tree (Figure 3B) were drawn with three groups' OTUs. The dilution curve (Figures 4A,B) constructed from the sequenced data has been basically stable, indicating that the sequenced data has been basically stable at this sequencing depth, and we have obtained the diversity of most of the microbiome contained in the samples.

**Figure 5:**
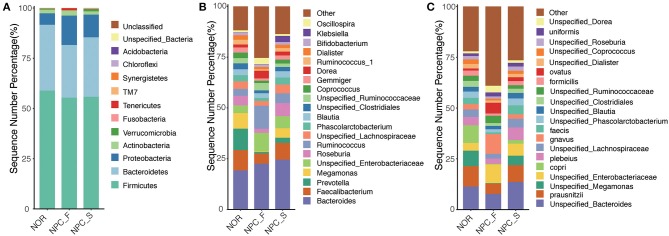
Comparison of relative taxa abundance among the healthy controls (NOR), familial NPC patients (NPC_F), and sporadic NPC patients (NPC_S) groups at phylum, genus and species levels. **(A)** The bar chart of relative taxa abundance among the three groups at phylum levels. **(B)** The bar chart of relative taxa abundance among the three groups at genus levels. **(C)** The bar chart of relative taxa abundance among the three groups at species levels.

At the phylum level for all species corresponding to the categorizable sequence, the main phylum is Firmicute, followed by Bacteroidete, Proteobacteria, Actinobacteria, and Verrucomicrobia (Figure 5A). Compared with the NOR group, the proportions of Proteobacteria in NPC_F (*P* = 0.0045) and NPC_S (*P* = 7.57E-05) were significantly increased. The distribution of the three groups at the genus and species levels is shown in (Figures 5B,C). It is found that the composition of the microorganisms in the three groups is distinctly different at different taxonomic levels.

#### Differences in Intestinal Flora Structure Between Groups

Based on the analysis of the DESeq2 method (Supplementary Table 3), the relative abundances between NPC_F and NOR were significantly different in 23 genus and 28 species; the relative abundances between NPC_S and NOR were significantly different in 9 genus and 17 species. And the relative abundance between NPC_F and NPC_S were significantly different in 13 genus and 22 species. Compared with the NOR group, the relative abundances of 8 genus such as *Clostridium, Veillonella*, and *Burkholderia* were significantly increased in NPC_F, while the relative abundances of 15 genus including *Akkermansia, Megamonas*, and *Roseburia* were significantly reduced in NPC_F. Compared with NOR, 8 species were significantly increased in NPC_F, such as *C. ramosum, Bacteroides fragilis, Citrobacter* spp. etc. While 20 species such as *A. muciniphila, Roseburia* spp., and *Gemmiger formicilis* were significantly reduced. In the NPC_S group, *Klebsiella, Veillonella, Clostridiaceae* spp., *Clostridium, Holdemania* etc., were increased significantly; at the species level, 14 species including *C. ramosum, Veillonella parvula, Veillonella dispar, Klebsiella* spp. were increased significantly in NPC_S, while *Mogibacteriaceae* spp., *Clostridiales* spp., *RF39* spp. etc., were reduced significantly. These differential genus and species can be used to construct a predictive model for the identification between familial NPC patients and sporadic NPC patients with healthy controls. Compared with NPC_S, *C. ramosum, Clostridium symbiosum, Blautia producta, Ruminococcus gnavus* etc., were significantly increased in NPC_F, while *Klebsiella* spp., *Prevotella stercorea* etc., were significantly increased in NPC_S.

From our current study, we evaluated the main intestinal flora commonly affected by diet, and found that some bacterium such as *Lactobacillus* spp. and *Faecalibacterium prausnitzii* can generate major metabolic byproducts including short chain fatty acid (73, 74); while *Enterococcus* spp., *Streptococcus* spp., and *Helicobacter pylori* were opportunistic pathogens in some cases (75–77), showing no significant difference between NPC patients and healthy volunteers. Nevertheless, some other bacterium may be affected by diet, like *Clostridium* spp., *Bacteroides* spp., *Roseburia* spp., *Eubacterium* spp., and *Escherichia coli* which were mainly rich in NPC, while *Bifidobacterium* spp., *Alistipes* spp., *Bilophila* spp., and *A. muciniphila* were mainly found in healthy volunteers. The above measures and findings inferred us that the influence of nutrition and diet on gut microbiota between the comparison of NPC and healthy people can be neglected.

**Figure 6:**
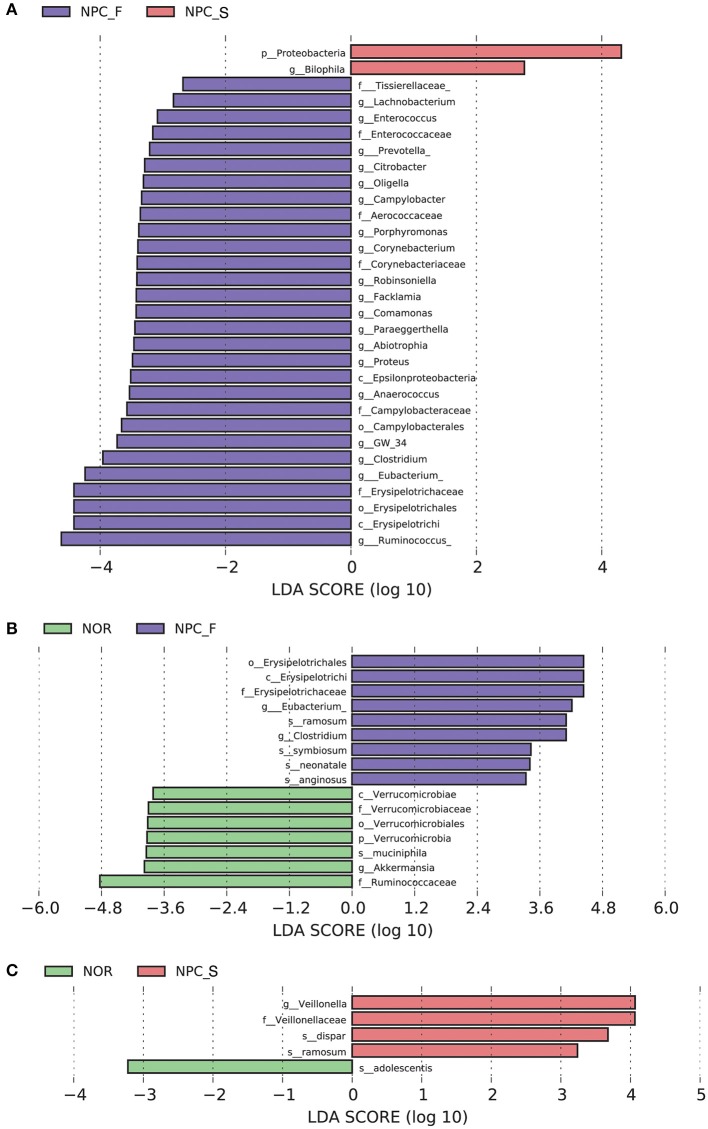
Characteristics of intestinal flora composition in the healthy controls (NOR), familial NPC patients (NPC_F), and sporadic NPC patients (NPC_S) groups. **(A)** Diagram of the LDA scores calculated at genus levels among NOR, NPC_F and NPC_S groups, enriched taxa of NPC_S are directed with a positive value (red), while enriched taxa of NPC_F are directed with a negative value (purple). Only the LDA score > 2 are shown in the figure. **(B)** Diagram of the LDA scores calculated at species level between NOR and NPC_F groups, enriched taxa of NPC_F are directed with a positive value (purple), enriched taxa of NOR are directed with a negative value (green). **(C)** Diagram of the LDA scores calculated at species level between NOR and NPC_S groups, enriched taxa of NPC_S are directed with a positive value (red), enriched taxa of NOR are directed with a negative value (green). (phylum to species: p, phylum; c, class; o, order; f, family; g, genus; s, species).

**Figure 7:**
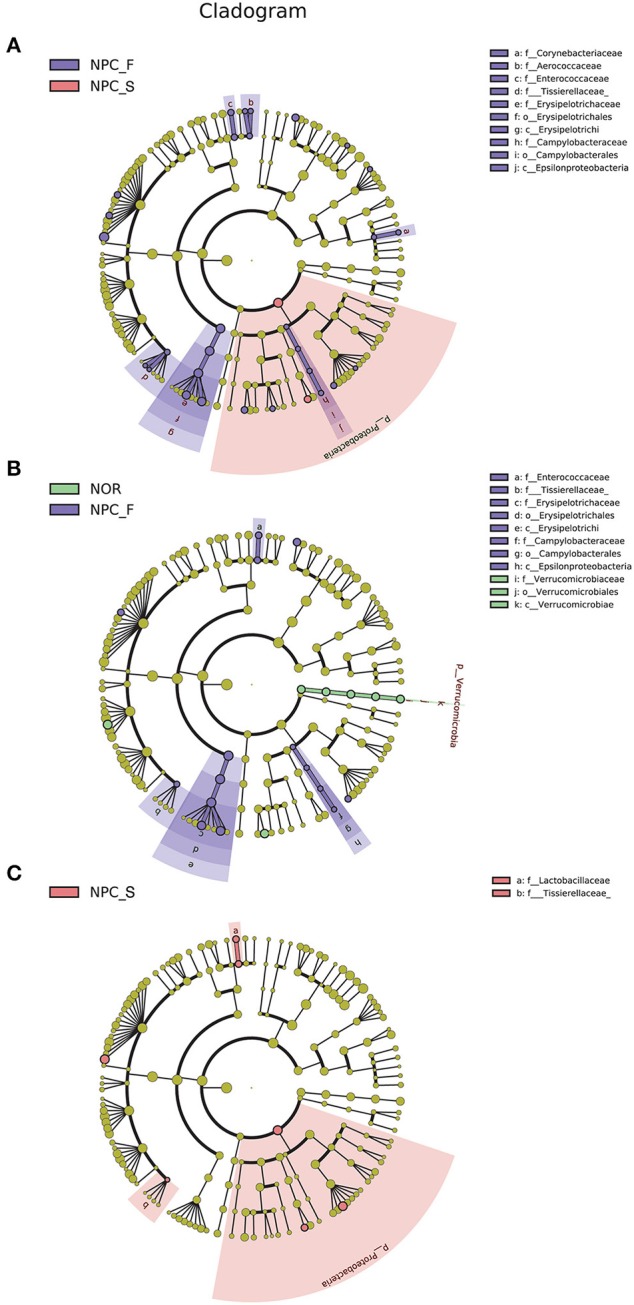
Enriched flora in the healthy controls (NOR), familial NPC patients (NPC_F), and sporadic NPC patients (NPC_S) groups are represented in a cladogram. The origin of the tree (bacteria) showed in the central point, the larger circles centered on the center represent decreasing species levels from phylum to genus (phylum to genus: p, phylum; c, class; o, order; f, family; g, genus). The relative abundance of the flora is showed by each circle's diameter. **(A)** LEfSe analysis cladogram of intestinal flora among NOR, NPC_F and NPC_S groups at genus level. **(B)** LEfSe analysis cladogram of intestinal flora between NOR and NPC_F at genus level. **(C)** LEfSe analysis cladogram of intestinal flora between NOR and NPC_S at genus level.

The results of LEfSe were shown in Figures 6, 7. Comparision among the three groups, *Clostridium, Eubacterium* etc., were increased significantly in NPC_F, and *Bilophila* increased significantly in NPC_S. At the species level, compared with NOR, it was found that *C. ramosum, C. symbiosum* were increased significantly in NPC_F, while *A. muciniphila* was significantly reduced in NPC_F; *C. ramosum* and *V. dispar* increased significantly while *B. adolescentis* decreased significantly in NPC_S.

**Figure 8:**
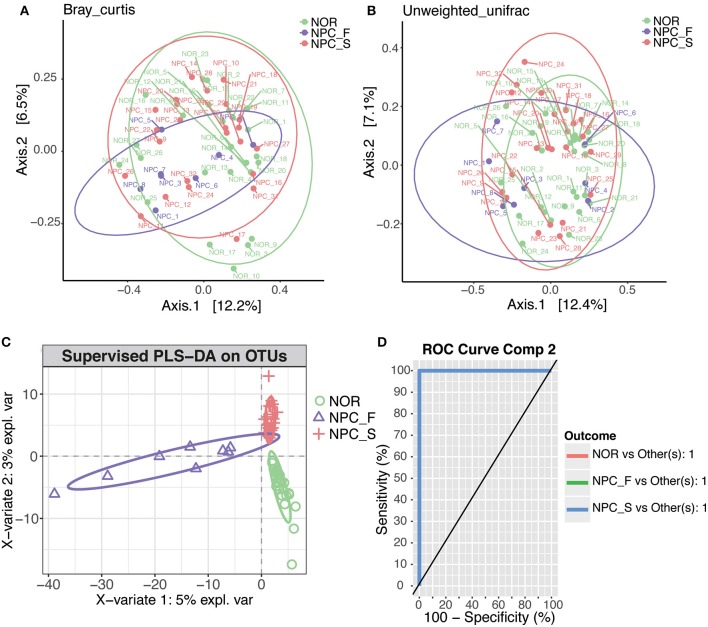
PCoA and PLS-DA analysis of the microbiome among the healthy controls (NOR), familial NPC patients (NPC_F), and sporadic NPC patients (NPC_S) groups, different points or patterns are on behalf of different samples, different groups are showed in different colors, each large circle represents a group. The degree of discrepancy of the microbial structure of the samples is showed by the distance between the points or patterns. **(A)** Bray-Curtis PCoA based on the relative abundance of OTU (99% similarity level), NOR: green points, NPC_F: purple points, NPC_S: red points. **(B)** Unweighted UniFrac PCoA based on the relative abundance of OTU (99% similarity level), NOR: green points, NPC_F: purple points, NPC_S: red points. **(C)** The PLS-DA analysis on OTUs among the NOR, NPC_F and NPC_S groups, NOR: green circles, NPC_F: purple triangles, NPC_S: red crosses. **(D)** ROC analysis for the predictive value of the predictive model constructed based on PLS-DA analysis. The AUCs of the NOR, NPC_F and NPC_S groups all are 1.

The first two main coordinates PC1 and PC2 with the largest contribution rate were obtained by using PCoA analysis (the explanatory variations were Bray-Curtis: 12.2 and 6.5% (Figure 8A), and Unweighted UniFrac: 12.4 and 7.1% (Figure 8B), respectively. Intestinal flora of NPC_F, NPC_S, and NOR groups was not completely clustered in the PCoA diagram.

According to the PLS-DA analysis (Figure 8C), the three distinct clusters indicated that the intestinal floras of the NPC_F, NPC_S, and NOR groups were clearly differentiated into three independent clusters, indicating that the composition of intestinal flora was significantly different among the three groups. All the AUCs were 1 (Figure 8D), suggesting that the prediction model established based on the detected differential genus was very good by using the PLS-DA analysis and that the intestinal flora related to the two groups of NPC were strong prediction factors and could be used as risk factors for NPC. For example, *C. ramosum* could be used as a strong prediction factor for NPC and is likely to be developed as a biomarker for high-risk populations of NPC and NPCs.

**Figure 9:**
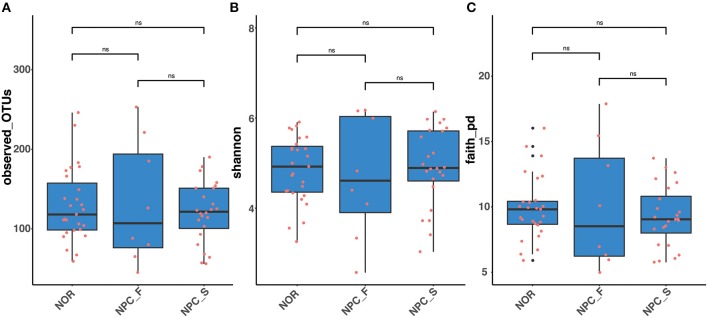
The comparision of Alpha diversity among the healthy controls (NOR), familial NPC patients (NPC_F), and sporadic NPC patients (NPC_S) groups based on different indices. **(A)** Observed_OTUs. **(B)** Shannon index. **(C)** Faith's phylogenetics diversity index. ns, no significance, Wilcox_Test.

**Figure 10:**
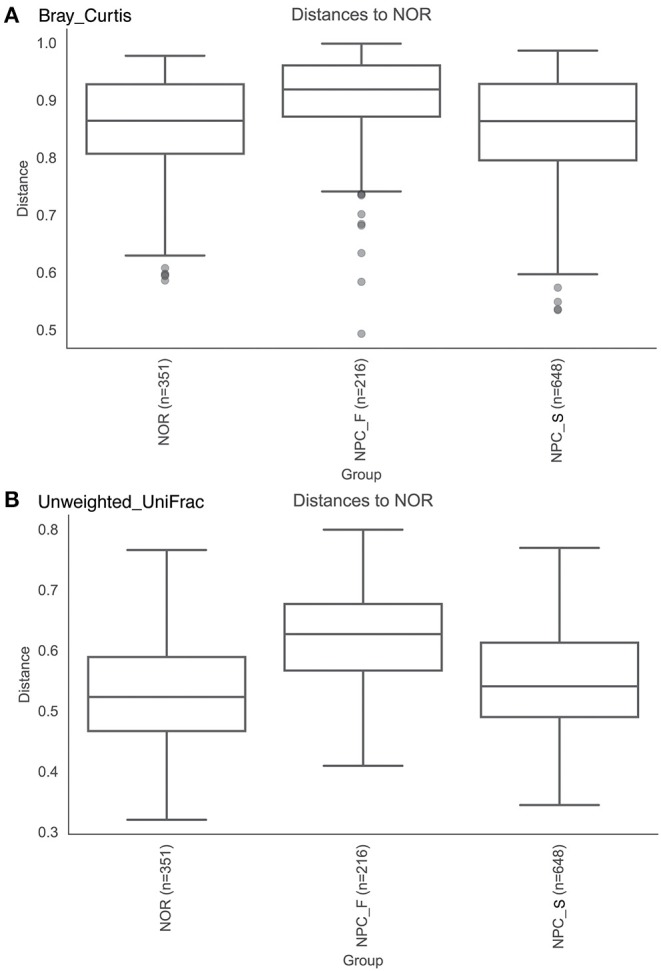
The comparision of Beta diversity among the healthy controls (NOR), familial NPC patients (NPC_F), and sporadic NPC patients (NPC_S) groups based on different indices. **(A)** Bray-Curtis distance index, Anosim. **(B)** Unweighted UniFrac distance index, Anosim.

The observed OTUs, shannon and faith's phylogenetic diversity indices of the intestinal flora between the three groups of NPC_F, NPC_S, and NOR were calculated, and the statistical analysis of alpha diversity between every two groups showed there was no significant difference (Figure 9, Supplementary Table 4). Statistical analysis of beta diversity between each two groups was conducted, based on Bray-Curtis distance index (Figure 10A, Supplementary Table 5), it was found that the differences in beta diversity between NPC_F and NPC_S (*P* = 0.018), NPC_F and NOR (*P* = 0.012), were significant from each other, while that of the NPC_S and NOR (*P* = 0.337) was not significant; based on Unweighted UniFrac distance index (Figure 10B, Supplementary Table 5), the beta diversity between NPC_F and NPC_S (*P* = 0.0045), NPC_F and NOR (*P* = 0.0045) were significantly different, while that of between NPC_S and NOR (*P* = 0.151) was not significantly different. The NPC_F, NPC_S, and NOR groups had no significant difference in the gut microbial abundance and diversity. However, the NPC_F group was significantly different from the NPC_S group and the NOR group in the gut microbial structure, indicating the microbial structure of familial NPC patients was significantly altered relative to both sporadic NPC patients and healthy controls.

#### Statistical Analysis of Correlation Between Intestinal Flora and Clinical Variables

**Figure 11:**
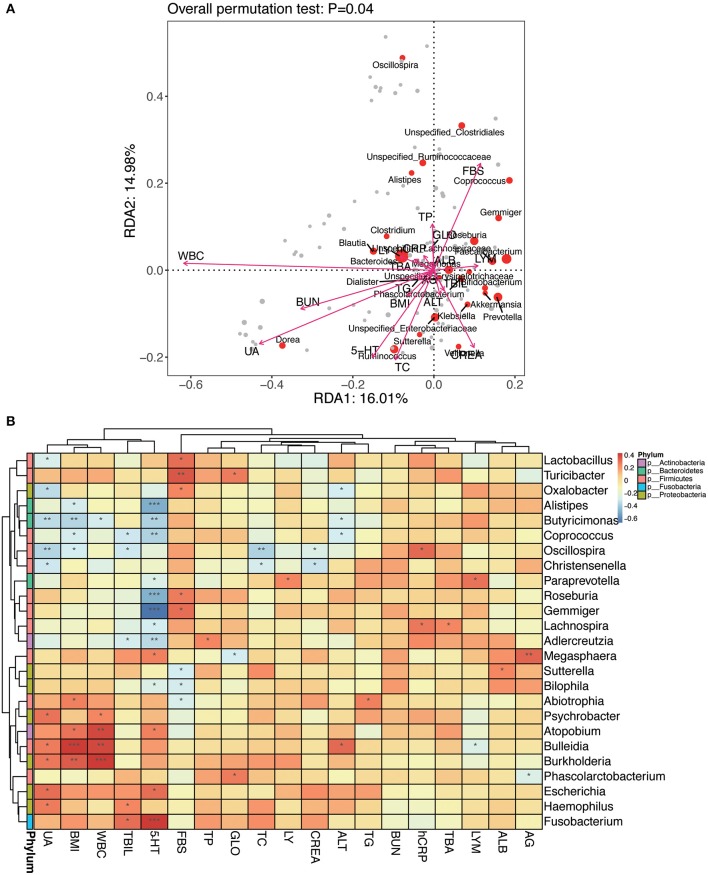
The relationship between intestinal flora and clinical variables at the genus level. **(A)** RDA ranking map. RDA1:16.01% and RDA2:14.98% represent the magnitude of the percentage of variance interpreted in the direction of the two axes, respectively; clinical variables were indicated by arrows, the length of an arrow represented the size of the variance between the clinical variable and the flora distribution. An acute angle between two arrows indicated a positive correlation between two clinical variables, and an obtuse angle indicated a negative correlation. Each point represented a species, and the larger the point, the more abundance of the species. **(B)** Heat map for Spearman correlation analysis between intestinal flora and clinical variables at the genus level. X-axis: clinical variables, Y-axis: genus. The branches of the figure on the left indicates the classification of phylum. *R*-values (rank correlations) are shown in different colors in the heat map, the figure on the right shows the color gradient corresponding to different *R*-value; *P* < 0.05 is showed in the figure. ^*^P < 0.05, ^**^P < 0.01, ^***^P < 0.001.

**Figure 12:**
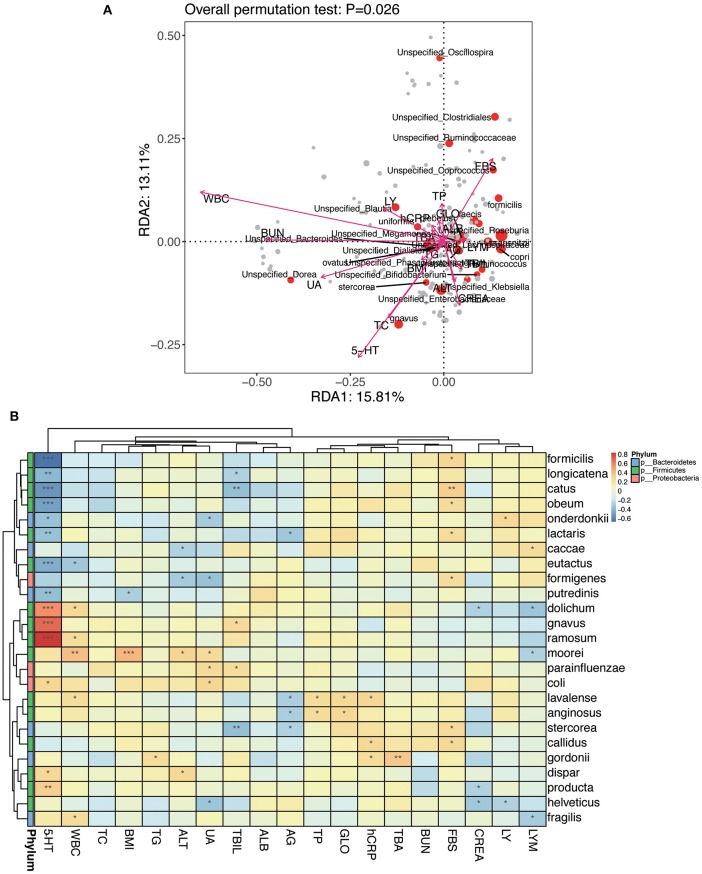
The relationship between intestinal flora and clinical variables at the species level. **(A)** RDA ranking map; RDA1:15.81% and RDA2:13.11% represent the magnitude of the percentage of variance interpreted in the direction of the two axes, respectively. **(B)** Heat map for Spearman correlation analysis between intestinal flora and clinical variables at the species level. ^*^P < 0.05, ^**^P < 0.01, ^***^P < 0.001.

An RDA ranking map (Figure 11A) and a correlation heat map (Figure 11B) of NPC_F, NPC_S, and NOR group between intestinal flora at the genus level and clinical variables. Similarly, an RDA ranking map (Figure 12A) and a correlation heat map (Figure 12B) of NPC_F, NPC_S, and NOR group between intestinal flora at the species level and clinical variables. Based on the RDA ranking map, the correlation between total of the clinical variables and the intestinal flora was significant (at genus level: *P* = 0.04, Figure 11A; at species level: *P* = 0.026, Figure 12A), with the most relevant variables such as WBC, UA, FBS, 5-HT, BUN, TC, CREA.

Based on the genus-level RDA ranking map (Figure 11A) and the correlation heat map (Figure 11B), *Oscillospira* has a strong correlation with several clinical variables: positively correlated with hCRP (*r*: 0.32, *P* = 0.012); negatively correlated with BMI (*r*: −0.30, *P* = 0.022), TC (*r*: −0.37, *P* = 0.0042), UA (*r*: −0.36, *P* = 0.0047), and weakly positively correlated with TBA (*r*: 0.17, *P* = 0.20). Based on the species-level RDA ranking map (Figure 12A) and the correlation heat map (Figure 12B), *C. ramosum* was positively correlated with 5-HT (*r*: 0.85, *P* = 2.81E-17), and *V. dispar* was positively correlated with ALT (*r*: 0.30, *P* = 0.020).

#### Species Interaction Network Analysis

**Figure 13:**
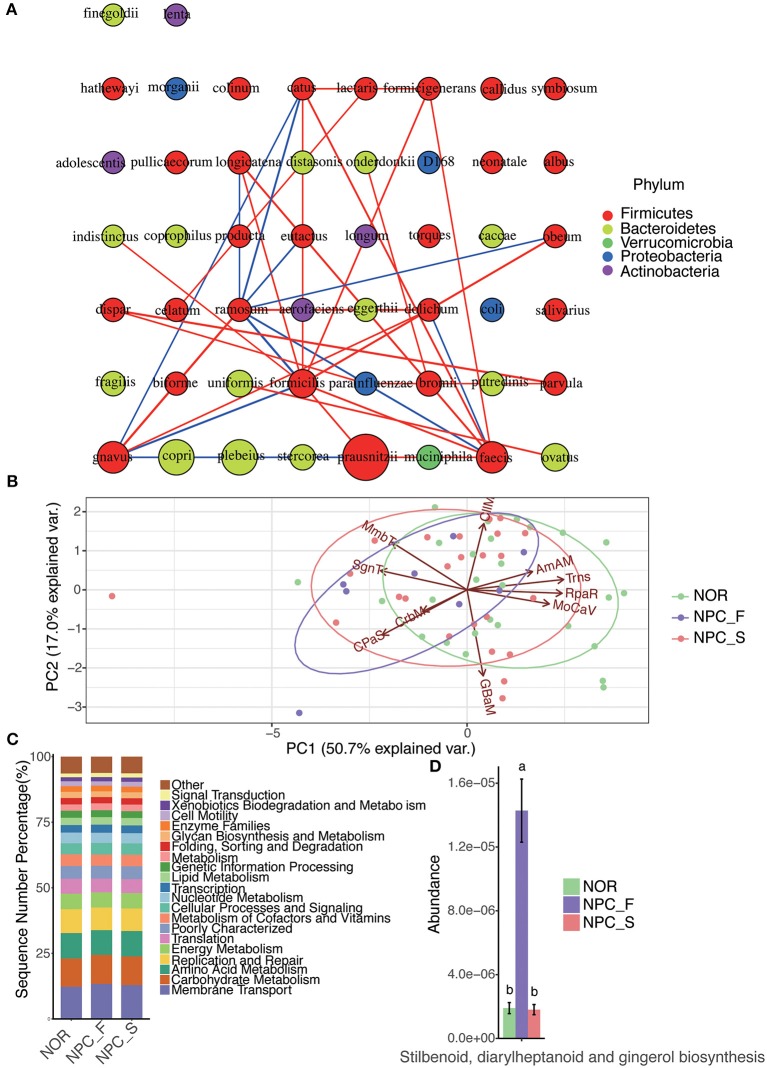
The intestinal flora interaction network analysis and functional PICRUSt analysis among the healthy controls (NOR), familial NPC patients (NPC_F) and sporadic NPC patients (NPC_S) groups. **(A)** The map of the species interaction network analysis; a circle represents a bacteria, the size of the circle represents its relative abundance, different color represents different classification at phylum level, the line between the circles represents the correlation between the two bacteria is significant (*P* < 0.05), the red color of the line represents a positive correlation, while the blue one represents the negative correlation, the line is more rough, corresponding correlation coefficient value is greater. **(B)** The diagram of PCA analysis for the predicted functions on the second KEGG-pathway level; The PC1-axis (50.7%) and PC2-axis (17.0%) represent the contribution of the two principal components to the sample difference are 50.7% and 17.0%, respectively, each point represents a sample, each circle represents a group (NOR in green, NPC_F in purple and NPC_S in red), and the arrow direction and length represent the direction and dominant ability of the prediction function in the group, respectively. (AmAM, Amino Acid Metabolism; CIIM, Cell Motility; CPaS, Cellular Processes and Signaling; CrbM, Carbohydrate Metabolism; GBaM, Glycan Biosynthesis and Metabolism; MmbT, Membrane Transport; MoCaV, Metabolism of Cofactors and Vitamins; RpaR, Replication and Repair; SgnT, Signal Transduction; Trns, Translation). **(C)** The bar chart of the predicted functions at the second KEGG-pathway level. **(D)** The bar chart of the abundance of the function of stilbenoid, diarylheptanoid and gingerol biosynthesis of the NOR, NPC_F and NPC_S groups, the value of a and b represent that there is significant difference in abundance of this function compared NPC_F with NOR, and NPC_F with NPC_S groups.

Figure 13A shows that *C. ramosum* is significantly associated with a variety of species, *C. ramosum* was positively correlated with opportunistic pathogens such as *R. gnavus* (*r*: 0.76, *P* = 2.72E-12), *Eubacterium dolichum* (*r*: 0.68, *P* = 9.06E-07), *C symbiosum* (*r*: 0.42, *P* = 0.016), and negatively correlated with the beneficial bacteria such as *Roseburia faecis* (*r*: −0.61, *P* = 3.08E-07) and *F. prausnitzii* (*r*: −0.49, *P* = 9.20E-05); which suggests that *C. ramosum* is probably a bacterium that affects the pathogenesis of NPC and provides us a direction for our future research on opportunistic pathogens and beneficial bacteria associated with *C. ramosum*. Meanwhile, *V. dispar* is positively correlated with *V. parvula* (*r*: 0.73, *P* = 5.52E-11) and *Haemophilus parainfluenzae* (*r*: 0.55, *P* = 5.13E-06).

#### Prediction of Intestinal Microbial Function

To determine whether the structural differences among the three groups of intestinal flora corresponded to functional changes, we used PICRUSt to perform a functional prediction analysis of the 16S sequences and performed a PCA analysis for the predicted functions (Figure 13B) and a comparative analysis of the predicted functions at the second KEGG-pathway level (Figure 13C). The comparison of enriched KEGG-pathway among NPC_F, NPC_S, and NOR groups by Dunn test is shown in the Supplementary Table 7. On the first KEGG-pathway level, the intestinal microbial function of NPC_F was found to be significantly correlated with HmnD (Human Diseases) (*P* = 0.080), the intestinal microbial function of the NOR group was significantly correlated with OrgS (Organismal Systems) (*P* = 0.014). On the second KEGG-pathway level, the gut microbial function of NPC_F was associated with neurodegenerative diseases (*P* = 0.023), lipid metabolism (*P* = 0.073); NPC_S was also associated with neurodegenerative diseases (*P* = 0.045); while the gut microbial function of NOR was mainly associated with immunity, digestion, endocrine system, energy, and nutrient digestion. At the same time, through Duncan test, it was found that the function of stilbenoid, diarylheptanoid, and gingerol biosynthesis in NPC_F was significantly increased (Figure 13D). The statement above shows that the predicted function of the intestinal flora can reflect the health status of the subjects. NPC patients are more susceptible to neurodegenerative diseases, and their intestinal function is more likely to be related to the synthesis of secondary metabolites and lipid metabolism; while the intestinal microbial function of the NOR group was mainly related to immunity, digestion, endocrine, energy, and nutrient digestion.

## Discussion

This study investigated the correlation between the changes in the intestinal flora and NPC by an examination of the intestinal flora and multiple clinical indicators of the blood of 8 carefully screened patients of familial NPC, 24 patients of sporadic NPC and 27 healthy controls and a comparison of the differences in their intestinal flora structures and biological functions. By analyzing the function of the intestinal floras of NPC patients, we aimed to provide a better biological marker for patients with familial and sporadic NPC and constructed a disease prediction model for high-risk populations.

In this study, we found that the intestinal microbiota structures of familial NPC patients and sporadic NPC patients were different from that of the healthy volunteers. At the phylum level, Proteobacteria were significantly increased in NPC_F and NPC_S. A previous study showed that the increased abundance of Proteobacteria was positively correlated with inflammatory diseases (78) such as nonalcoholic fatty liver disease (NAFLD) (79, 80), colitis (81, 82), inflammatory bowel diseases (IBD) (83–85), Crohn's disease (CD) and ulcerative colitis (UC) (86), and asthma and chronic obstructive pulmonary disease (COPD) (87, 88). The Proteobacteria in the mouth are significantly associated with the severity of mucositis in patients with NPC (89). Some bacterial components (such as Lipopolysaccharide, LPS) in the opportunistic pathogenic bacteria of the Proteobacteria phylum may produce pro-inflammatory factors that can cause cancer through activating the host's pattern-recognition receptors (such as Toll-like receptors, TLRs) (90).

*C. ramosum* and opportunistic pathogens such as *Citrobacter* spp., *Veillonella* spp., *Prevotella* spp., *Campylobacter* spp. in the intestinal flora of familial NPC patients were significantly increased, while the abundances of the anti-inflammatory bacteria *A. muciniphila*, and butyrate-producing bacteria *Roseburia* spp. were significantly decreased. *C. ramosum, V. parvula, V. dispar*, and *Klebsiella* spp. were significantly increased in sporadic NPC patients, and the abundance of the probiotic *B. adolescentis* was significantly decreased.

Compared to the NOR group, the *C. ramosum* in both of the NPC_F and NPC_S groups was significantly increased. Using ELISA, we found that the levels of 5-HT in the sera of patients in the NPC_F and NPC_S groups were significantly higher than those in the NOR group, and *C. ramosum* was significantly associated with 5-HT. *C. ramosum* is a spore-forming, indigenous intestinal bacteria, whose metabolites can irritate ECs to secret 5-HT (46). It was found that *C. ramosum* is an important opportunistic pathogen in clinical (91–93). At the same time, several studies have discovered that some of the metabolites of the spore-forming microbiota from human gastrointestinal flora can modulate the elevation of 5-HT from ECs (30, 94, 95). More than 90% of 5-HT in the human body is biosynthesized by ECs, which plays a crucial role in human physiological function through activation of the different 5-HT receptors on different kinds of cells, such as intestinal epithelial cells (96), platelets (97) and immune cells (98). In addition, it was discovered that there are many kinds of 5-HTR subtypes on a variety of cancers, including PC (32), HCC (33–35), CRC (36) etc. 5-HT could promote the development and progression of these cancers by activating the 5-HTR subtypes, such as 5-HT1A, 5-HT1B, 5-HT2A, 5-HT2B (44, 99). 5-HT1B was overexpressed in human NPC samples (44), which reminded us that the elevated 5-HT in plasma in NPC could promote the progression of NPC through 5-HTR. These findings suggest that the significantly elevated *C. ramosum* in the intestinal flora of NPC patients may affect the development and progression of NPC through increasing the 5-HT levels in the blood. We also found that both the *C. ramosum* and 5-HT were significantly higher in the NPC_F group than those in the NPC_S group, and this outcome may have occurred because NPC_F patients had family histories of NPC.

In the human body, *A. muciniphila* is an anti-inflammatory probiotic (100) that exerts beneficial effects on the host through regulating the mucin metabolism and immune responses, and *A. muciniphila* also shapes the composition of the host intestinal flora (101). Previous studies have found that reduced *A. muciniphila* levels are associated with the development and progression of many malignant tumors such as CRC and breast cancer and that *A. muciniphila* has a positive effect on the response of tumors to chemotherapeutic drugs and immune checkpoint inhibitors and improves the intestinal microbial composition (102, 103). Our study found that *A. muciniphila* was significantly reduced in patients with familial NPC. Therefore, we hypothesized that the reduction of *A. muciniphila* in patients with familial NPC lead to decreased levels of mucin metabolites beneficial to the host, which disrupts the homeostasis of the barrier function and immune function of the host intestine and eventually triggers NPC.

*Roseburia* spp. was significantly reduced in patients with familial NPC. *Roseburia* spp. is one of the main bacteria that generate the butyrate required by the human body that suppresses the growth and proliferation of tumor cells through multiple pathways (104–106). One study showed that the siblings of CD patients had similar disruptions of intestinal flora as CD patients, manifested by a low abundance of *Roseburia* spp. (107), suggesting that *Roseburia* spp. is familial. We believe that the familial nature of *Roseburia* spp. is probably related to the familial nature of NPC, which may explain the significant reduction in the butyrate supply in patients with familial NPC due to the significant reduction of butyrate-producing *Roseburia* spp. in their intestinal flora. The reduced butyrate may lead to an abnormal expression of genes related to NPC in the body and an enhanced Warburg effect of cancer cells, which then promotes tumor development and progression.

*V. parvula* and *V. dispar* were significantly increased in patients with sporadic NPC. NPC patients often have clinical symptoms such as cervical lymph node enlargement, nasal congestion and blood stasis. A previous study found that *Veillonella* spp. was significantly increased in CD patients (108). *V. parvula* is associated with many inflammatory diseases such as endocarditis (109), meningitis (110), and bacteremia (111). *V. dispar* is significantly increased in autoimmune hepatitis, and it was found that this bacterium was positively correlated with the serum AST level and the extent of disease activity (112). ALT and AST both reflect liver damage, with ALT being more sensitive. Our study found that *V. dispar* was positively correlated with ALT. *Klebsiella* spp. is significantly increased in sporadic NPC, and a previous study showed that *Klebsiella* spp. is one of the most common bacteria that causes infections in tumor patients (113). *Klebsiella* spp. is the main bacterium in the oral flora during the immunosuppression of cancer patients, which may lead to severe local or systemic disease in these patients (114). We believe that the changes in the intestinal flora of patients with sporadic NPC can lead to the disruption of the immune function by promoting inflammation that triggers cancer and may also affect the liver function. Therefore, special attention should be paid to the liver function during diagnosis and treatment. We found that *B. adolescentis* was significantly reduced in patients with sporadic NPC. *Bifidobacterium* is a probiotic that is mainly found in the intestine of breastfed infants (115), and this bacterium can metabolize dietary fiber to produce acetate and lactate, which can be further metabolized to produce propionate and butyrate (116, 117). Propionate and butyrate are beneficial to the physiological function of various tissues and organs *in vivo* (118). Previous studies have found that *Bifidobacterium* has an anti-inflammatory effect *in vivo* (119–122), and several studies have shown that the substances produced by *B. adolescentis* have anti-inflammatory activity (123–125). In summary, the increases in the pro-inflammatory bacteria *C. ramosum, V. parvula*, and *V. dispar* and the reduction of the anti-inflammatory bacterium *B. adolescentis* in patients with sporadic NPC may lead to the physiological dysfunction of the body by inducing an inflammatory status that promotes tumor formation.

The relative abundances of *Oscillospira* in the NPC_F and NPC-S groups were increased, and *Oscillospira* was positively correlated with hCRP, negatively correlated with the body-mass index (BMI) and weakly and positively correlated with TBA. *Oscillospira* is an anaerobic bacterium whose abundance is negatively correlated with BMI (126, 127) and is significantly reduced in CD (128) and NAFLD (129). These findings all suggest that *Oscillospira* not only reduces the BMI of the host, but the reduction in its abundance is positively correlated with inflammation. *Oscillospira* is the only genus that increased in the cecum during fasting in mammals (130). Compared to vegetarian diets, the TBA and the abundances of *Oscillospira* and anti-biliary bacterium were increased in the intestine of dietary intervened carnivorous volunteers (131). It has also been found that the abundance of *Oscillospira* was significantly increased by more than 4 times (*P* = 0.041) in the intestine of patients with cholelithiasis, (132) and it is highly likely that *Oscillospira* plays an anti-TBA role. This study showed that the abundance of *Oscillospira* was increased in NPC patients, which might be related to a progressive decline in appetite during the disease. Red meat consumption is associated with an increased risk of NPC (133) that further promotes the increase of *Oscillospira* by increasing bile acid levels. Opportunistic pathogens can elevate inflammatory factors through microbe-related molecular patterns (MAMPs), affecting the homeostasis of the body's immune system, which, in turn, induces cancer (134). *Oscillospira* may promote the elevation of hCRP levels through MAMPs.

## Conclusion

The increase in the *C. ramosum* bacteria that promote the secretion of 5-HT is likely a key feature of the intestinal flora of human NPC patients. It is an urging issue to proceed additional studies with animal models, even with human models, to elaborate the mechanisms underlying the association among 5-HT, intestinal flora and NPC, with which we can manipulate the intestinal flora to screen, guard against and remedy NPC. The increases in *C. ramosum* and the opportunistic pathogens *Citrobacter* spp. and *Veillonella* spp. as well as the reduction of the anti-inflammatory bacteria *A. muciniphila* and butyrate-producing bacteria *Roseburia* spp. are key features of the intestinal flora of patients with familial NPC. The increases in *C. ramosum* and the pro-inflammatory bacteria *V. parvula* and *V. dispar* and the reduction in the anti-inflammatory bacteria *B. adolescentis* are probably characteristics of the intestinal flora of patients with sporadic NPC. Based on these characteristics, we can establish a predictive model for the intestinal flora of familial and sporadic NPCs, in which *C. ramosum*, a strong risk factor for NPC, may be used as a new biomarker for NPC patients. Based on this predictive model, we are likely to predict the disease risk in the population with a high risk of NPC and perform a non-invasive early screening for NPC using the biomarkers. However, this study is restricted by the limited number of enrolled patients. Based on the International Agency for Research on Cancer, the new cases of NPC only holding 0.7% of all diagnosed cancers in 2018 (1, 2). The incidence rate of NPC is 3.0/100 000 in China to 0.4/100 000 in white population (1, 2). The morbidity of NPC has been descending increasingly in recent years, the main decrement is from east and southeast Asia (135). The Third Xiangya Hospital is large and international hospital, from which we have got most of the cases based on the laboratory department. Considering the limited sample size, we also colaborated with other large hospitals, such as Hunan cancer hospital, to embrace more cases. Each of these cases has been carefully filtrated before being recruiting, and they are representative and precious. The discovery of this study also provided us a direction for our future research. Nevertheless, more studies with a larger number of NPC participants are needed to confirm this predictive model before being used in clinical. Through our findings, we may be able to monitor confirmed NPC patients and their intestinal flora and clinical indicators in the late disease stages and determine the relationship among the disease status, intestinal flora and clinical monitoring indicators, which may provide individualized methods to prevent and treat NPC.

## Data Availability Statement

The DNA sequencing data in this article is deposited in the NCBI BioProject database (https://www.ncbi.nlm.nih.gov/bioproject), with the accession number: PRJNA565548.

## Ethics Statement

This study was conducted as per the recommendations of the Human Specimen Study guidelines of the Institutional Review Board (IRB) of the Third Xiangya Hospital, Central South University, with written informed consent taken from all subjects. All subjects gave written informed consent in accordance with the Declaration of Helsinki. The protocol was approved by the IRB of the Third Xiangya Hospital, Central South University. The IRB number is 2019-S478.

## Author Contributions

HJ, JL, BZ, RH, JZ, ZC, XS, and XL: sample collection. HJ: detected samples, organized data, and wrote the article. XN: designed experiment. All authors have read and critically revised the manuscript.

### Conflict of Interest

The authors declare that the research was conducted in the absence of any commercial or financial relationships that could be construed as a potential conflict of interest.
