# Supplementary material for: Intestinal Flora Disruption and Novel Biomarkers Associated With Nasopharyngeal Carcinoma
Source: Front Oncol. 2019 Dec 6;9:1346. doi: 10.3389/fonc.2019.01346 (PMC6908496; doi:10.3389/fonc.2019.01346)
Supplement: Supplementary file 1 [file Table_1.docx]

Supplementary Table 1 Demographics of the Familial NPC, Sporadic NPC and Normal groups

|  | Familial NPC（NPC_F） | Sporadic NPC（NPC_S） | Normal（NOR） | P-value （NPC_F&NOR） | P-value (NPC_S&NOR) | | P-value (NPC_F&NPC_S) |
| --- | --- | --- | --- | --- | --- | --- | --- |
| Age(mean, SD) | 46.4,6 | 47.3,7.9 | 47.2,8.6 | 0.81 | 0.95 | 0.76 | |
| Sex(n, %) |  |  |  |  | | | |
| Female | 1,12.5% | 7,29.2% | 7,25.9% | 0.65 | >0.9999 | 0.64 | |
| Male | 7,87.5% | 17,70.8% | 20,74.1% |  |  |  |  |
| Race(n, %) |  |  |  |  | | | |
| Asian | 8,100% | 24,100% | 27,100% | 1 | | | |
| Ever smoker(n, %) | 2,25% | 11,45.8% | 6,22.2% | 0.1769 | | | |
| Ever drinker(n, %) | 1,12.5% | 4,16.7% | 2,7.4% | 0.59 | | | |
| First degree relative with other cancer (n, %) | 0 | 0 | 0 | 1 | | | |

Supplementary Table 2 The detective results and statistical analysis of clinical indicators

| Characteristics | Abbreviation | NPC_F | NPC_S | NOR | Unit | F Value | P Value (NOR:NPC_F:NPC_S) | P Value (NPC_F:NOR) | P Value (NPC_S:NOR) | P Value (NPC:NOR) |
| --- | --- | --- | --- | --- | --- | --- | --- | --- | --- | --- |
| Hypersensitive C-reactive protein | hCRP | 3.94±2.61 | 5.10±2.86 | 1.36±0.33 | mg/L | 4.467 | 0.016 | 0.11 | 0.022 | 0.0047 |
| White blood cells | WBC | 7.36±2.22 | 6.40±0.55 | 6.31±0.60 | ×10^9^/L | 1.331 | 0.27 | 0.56 | 0.91 | 0.66 |
| Lymphocytes | LY | 2.12±0.64 | 1.65±0.20 | 2.10±0.20 | ×10^9^/L | 5.165 | 0.0087 | 0.91 | 0.0023 | 0.023 |
| Percentage of lymphocytes | LYM | 29.54%±6.14% | 26.87%±3.93% | 33.96%±2.76% |  | 4.958 | 0.01 | 0.33 | 0.005 | 0.0058 |
| Creatinine | CREA | 72.85±8.08 | 64.86±4.17 | 79.15±6.06 | μmol/L | 8.072 | 0.00083 | 0.41 | 0.00037 | 0.0014 |
| Total cholesterol | TC | 4.34±1.09 | 4.54±0.40 | 4.93±0.31 | mmol/L | 1.748 | 0.18 | 0.31 | 0.0069 | 0.035 |
| Triglyceride | TG | 1.50±0.49 | 1.97±0.25 | 1.46±0.33 | mmol/L | 3.42 | 0.04 | 0.82 | 0.027 | 0.049 |
| Total protein | TP | 74.26±5.04 | 76.51±1.97 | 74.20±1.50 | g/L | 1.87 | 0.16 | 0.89 | 0.09 | 0.14 |
| Albumin | ALB | 46.44±2.10 | 46.23±1.41 | 47.9±0.85 | g/L | 2.69 | 0.077 | 0.043 | 0.08 | 0.017 |
| Globulin | GLO | 27.83±4.79 | 30.25±1.60 | 25.5±2.10 | g/L | 6.12 | 0.004 | 0.0014 | 0.23 | 0.0015 |
| Albumin ratio | AG | 1.73±0.30 | 1.56±0.11 | 1.87±0.11 |  | 7.81 | 0.001 | 0.00035 | 0.15 | 0.0004 |
| Total bilirubin | TBIL | 15.33±4.69 | 13.55±1.36 | 14.18±2.15 | μmol/L | 0.45 | 0.64 | 0.39 | 0.57 | 0.99 |
| Blood urea nitrogen | BUN | 5.40±1.72 | 4.59±0.47 | 4.79±0.45 | mmol/L | 1.19 | 0.31 | 0.57 | 0.37 | 1 |
| Uric acid | UA | 336.14±87.84 | 296.63±60.43 | 319.89±27.11 | μmol/L | 0.5 | 0.61 | 0.39 | 0.8 | 0.58 |
| Alanine aminotransferase | ALT | 22.33±5.60 | 48.62±16.64 | 24.93±4.53 | U/L | 5.99 | 0.0044 | 0.49 | 0.0075 | 0.011 |
| Fasting blood glucose | FBS | 5.13±0.60 | 5.46±0.36 | 4.7±0.19 | mmol/L | 7.75 | 0.001 | 0.00066 | 0.18 | 0.00037 |
| Total bile acid | TBA | 3.64±1.63 | 5.43±1.56 | 2.65±0.76 | μmol/L | 6.33 | 0.0033 | 0.0053 | 0.24 | 0.0023 |
| 5-hydroxytryptamine | 5-HT | 3.81±0.85 | 3.00±0.21 | 2.42±0.30 | ng/mL | 12.85 | 2.55411E-05 | 0.0046 | 0.0014 | 0.00032 |

Supplementary Table 4 Statistical analysis of Alpha diversity based on observed OTUs, shannon index and faith's phylogenetics diversity(faith_pd) index (Kruskal_Wallis)

| Alpha Diversity Index | Group | H-value | P-value | Q-value |
| --- | --- | --- | --- | --- |
| Observed_OTUs | NPC_F:NOR | 0.125 | 0.724 | 0.913 |
|  | NPC_S:NOR | 0.039 | 0.843 | 0.913 |
|  | NPC_F:NPC_S | 0.012 | 0.913 | 0.913 |
| Shannon | NPC_F:NOR | 1.42E-14 | 1 | 1 |
|  | NPC_S:NOR | 0.279 | 0.597 | 1 |
|  | NPC_F:NPC_S | 0.030 | 0.862 | 1 |
| Faith_pd | NPC_F:NOR | 0.099 | 0.753 | 0.931 |
|  | NPC_S:NOR | 0.890 | 0.345 | 0.931 |
|  | NPC_F:NPC_S | 0.008 | 0.931 | 0.931 |

Supplementary Table 5 Statistical analysis of Beta diversity based on Bray-Curtis and

Unweighted UniFrac index (Anosim)

| Beta Index | Group | R-value | P-value | Q-value |
| --- | --- | --- | --- | --- |
| Bray-Curtis | NPC_F:NOR | 0.317 | 0.004 | 0.012 |
|  | NPC_S:NOR | 0.008 | 0.337 | 0.337 |
|  | NPC_F:NPC_S | 0.260 | 0.012 | 0.018 |
| Unweighted UniFrac | NPC_F:NOR | 0.494 | 0.002 | 0.0045 |
|  | NPC_S:NOR | 0.024 | 0.151 | 0.151 |
|  | NPC_F:NPC_S | 0.323 | 0.003 | 0.0045 |

Supplementary Table 6 Tumor stage of recruited NPC (84), and familial NPC (8) and sporadic NPC (24) patients included in the study

| Tumor Stage | Recruited NPC(84) | Included Familial NPC(8) | Included Sporadic NPC(24) |
| --- | --- | --- | --- |
| Stage 2 | 8 | 2 | 3 |
| Stage 3 | 44 | 5 | 15 |
| Stage 4 | 32 | 1 | 6 |
